# Supplementary material for: Modelling Skylarks (Alauda arvensis) to Predict Impacts of Changes in Land Management and Policy: Development and Testing of an Agent-Based Model
Source: PLoS One. 2013 Jun 6;8(6):e65803. doi: 10.1371/journal.pone.0065803 (PMC3675089; doi:10.1371/journal.pone.0065803)
Supplement: Supporting Information S4 — The skylark ODdox as a zipped archive. (ZIP) [file pone.0065803.s004.zip › Skylark_ODdox/class_skylark___adult.html]

ALMaSS Skylark ODdox: Skylark\_Adult Class Reference


ALMaSS Skylark ODdox
 2.0


- Main Page
- Related Pages
- Classes
- Files

- Class List
- Class Index
- Class Hierarchy
- Class Members

Public Member Functions |
Public Attributes |
Protected Member Functions |
Protected Attributes

Skylark\_Adult Class Reference

`#include <skylarks_all.h>`

List of all members.

|  |  |
| --- | --- |
| Public Member Functions | |
| virtual void | CopyMyself (int a\_sktype) |
|  | Skylark\_Adult (int x, int y, double size, int age, SkTerritories \*Terrs, Landscape \*L, Skylark\_Population\_Manager \*SPM, int bx, int by, int mh) |
|  | ~Skylark\_Adult () |
| Public Member Functions inherited from Skylark\_Base | |
| void | AddStriglingMort (int lifestage) |
| bool | InSquare (rectangle R) |
| virtual double | On\_FoodSupply (double) |
|  | Skylark\_Base (int x, int y, SkTerritories \*Terrs, Landscape \*L, Skylark\_Population\_Manager \*SPM, int bx, int by, int mh) |
| virtual int | WhatState () |
| Public Member Functions inherited from TAnimal | |
| virtual void | BeginStep (void) |
| void | CheckManagement (void) |
| void | CheckManagementXY (int x, int y) |
| virtual void | CopyMyself () |
| virtual void | Dying () |
| virtual void | EndStep (void) |
| virtual void | KillThis () |
| virtual bool | OnFarmEvent (FarmToDo) |
| void | SetX (int a\_x) |
| void | SetY (int a\_y) |
| virtual void | Step (void) |
| int | Supply\_m\_Location\_x () |
| int | Supply\_m\_Location\_y () |
| unsigned | SupplyFarmOwnerRef () |
| int | SupplyPolygonRef () |
| AnimalPosition | SupplyPosition () |
|  | TAnimal (int x, int y, Landscape \*L) |
| Public Member Functions inherited from TALMaSSObject | |
| void | OnArrayBoundsError () |
|  | TALMaSSObject () |
| virtual | ~TALMaSSObject () |

|  |  |
| --- | --- |
| Public Attributes | |
| bool | Paired |
| Public Attributes inherited from Skylark\_Base | |
| int | Age |
| int | m\_Born\_x |
| int | m\_Born\_y |
| TTypesOfSkState | m\_CurrentSkState |
| int | m\_MyHome |
|  | The vegetation type where the skylark was born. |
| Skylark\_Population\_Manager \* | m\_OurPopulationManager |
| SkTerritories \* | m\_OurTerritories |
| double | m\_pcide\_conc |
| double | m\_pesticide\_accumulation |
| double | m\_Size |

|  |  |
| --- | --- |
| Protected Member Functions | |
| bool | GetBadWeather () |
|  | Extreme weather conditions check. |
| double | GetVegHindrance (int PolyRef) |
| double | GetWeatherHindrance () |
| virtual double | RemoveEM (double food) |
| Protected Member Functions inherited from Skylark\_Base | |
| bool | DailyMortality (int mort) |
| virtual void | PesticideResponse () |
| Protected Member Functions inherited from TAnimal | |
| void | CorrectWrapRound (void) |
|  | Corrects wrap around co-ordinate problems. |

|  |  |
| --- | --- |
| Protected Attributes | |
| bool | BSuccess |
| int | GoodWeather |
| vector< Point > \* | m\_aTerrlist |
| double | MyExtractEff |
| skTerritory\_struct | MyTerritory |

---

## Constructor & Destructor Documentation

|  |  |  |  |
| --- | --- | --- | --- |
| Skylark\_Adult::Skylark\_Adult | ( | int | *x*, |
|  |  | int | *y*, |
|  |  | double | *size*, |
|  |  | int | *age*, |
|  |  | SkTerritories \* | *Terrs*, |
|  |  | Landscape \* | *L*, |
|  |  | Skylark\_Population\_Manager \* | *SPM*, |
|  |  | int | *bx*, |
|  |  | int | *by*, |
|  |  | int | *mh* |
|  | ) |  |  |

References Skylark\_Base::Age, BSuccess, GoodWeather, m\_aTerrlist, Skylark\_Base::m\_Size, MeanExtractionRatePerMinute, MyExtractEff, MyTerritory, Paired, random(), and skTerritory\_struct::ref.

: Skylark\_Base( x, y, Terrs, L, SPM, bx, by, mh ) {

m\_Size = size;

Age = age;

BSuccess = false;

MyExtractEff = ( 0.90 + double( random( 21 ) / 100.0 ) ) \* MeanExtractionRatePerMinute;

GoodWeather = 0;

Paired = false;

MyTerritory.ref = -1;

m\_aTerrlist = new vector<Point>;

}

|  |  |  |  |  |
| --- | --- | --- | --- | --- |
| Skylark\_Adult::~Skylark\_Adult | ( |  | ) |  |

References m\_aTerrlist.

{

delete m\_aTerrlist;

}

---

## Member Function Documentation

|  |  |  |  |  |  |
| --- | --- | --- | --- | --- | --- |
| virtual void Skylark\_Adult::CopyMyself | ( | int | *a\_sktype* | ) |  |

virtual

Referenced by Skylark\_Population\_Manager::Catastrophe().

|  |  |  |  |  |
| --- | --- | --- | --- | --- |
| bool Skylark\_Adult::GetBadWeather | ( |  | ) |  |

protected

Extreme weather conditions check.

Referenced by Skylark\_Female::st\_Arriving(), Skylark\_Male::st\_Arriving(), Skylark\_Female::st\_BuildingUpResources(), Skylark\_Female::st\_Finding\_Territory(), Skylark\_Female::st\_Floating(), Skylark\_Male::st\_Floating(), Skylark\_Female::st\_Flocking(), Skylark\_Male::st\_Flocking(), Skylark\_Female::st\_TempLeavingArea(), and Skylark\_Male::st\_TempLeavingArea().

{

//return m\_OurPopulationManager->IsBadWeather();

return false;

}

|  |  |  |  |  |  |
| --- | --- | --- | --- | --- | --- |
| double Skylark\_Adult::GetVegHindrance | ( | int | *PolyRef* | ) |  |

protected

References TAnimal::m\_OurLandscape, Landscape::SupplyHasTramlines(), Landscape::SupplySkScrapes(), Landscape::SupplyVegDensity(), Landscape::SupplyVegHeight(), Landscape::SupplyVegPatchy(), CfgFloat::value(), VegHindranceD, and VegHindranceH.

Referenced by Skylark\_Male::GetFood().

{

#ifdef \_\_CJTDebug\_5

if ( IsAlive() != 0x0DEADC0DE ) DEADCODEError();

#endif

/\* CJT Changed in favour of a function of veg density double veg\_biomass = m\_OurLandscape->SupplyVegBiomass(polyref);

double veg\_height = m\_OurLandscape->SupplyVegHeight(polyref); int bio = (int)floor(0.5+veg\_biomass/(VeryHighDensityVeg/

double(HindranceBiomassCategories))); // 0-24, 25-49, 50-74,74-100 categories

int hei=(int)floor(0.5+veg\_height/(100.0/double(HindranceHeightCategories)));

if (hei>HindranceHeightCategories-1) hei=HindranceHeightCategories-1;

if (bio>HindranceBiomassCategories-1) bio=HindranceBiomassCategories-1; return VegHindrance[hei][bio]; \*/

if ( m\_OurLandscape->SupplyVegPatchy( polyref ) ) return 1.0;

int veg\_height = ( int )floor( 0.5 + m\_OurLandscape->SupplyVegHeight( polyref ) );

if ( veg\_height < 26 ) return 1.0;

int veg\_dens = m\_OurLandscape->SupplyVegDensity( polyref );

// double vh = ( VegHindranceH[ veg\_height ] \* 2 + VegHindranceD[ veg\_dens ] ) / 3.0;

//double vh = (0.33\*( DensityScore[ veg\_dens ] + 2 \* HeightScore[ ( int )veg\_height ] ) \* 0.33333333)+0.67;

double vh = ( VegHindranceD[ veg\_dens ] + 2 \* VegHindranceH[ veg\_height ] ) \* 0.3333;

#ifdef \_\_TRAMLINES\_ON

// Tramlines affect the amount of veg hindrance only

// vh must be increased by a factor corresponding to the tramline effect

// IF USING SKLARK SCRAPES !

if ( m\_OurLandscape->SupplySkScrapes( polyref )) {

vh += ( ( 1.0 - vh ) \* cfg\_tramline\_foraging.value() );

}

else if ( m\_OurLandscape->SupplyHasTramlines( polyref )) {

vh += ( ( 1.0 - vh ) \* cfg\_tramline\_foraging.value() );

}

#endif

return vh;

}

|  |  |  |  |  |
| --- | --- | --- | --- | --- |
| double Skylark\_Adult::GetWeatherHindrance | ( |  | ) |  |

protected

References TAnimal::m\_OurLandscape, RainHindrance, Landscape::SupplyRain(), Landscape::SupplyTemp(), and TempHindrance.

Referenced by Skylark\_Male::GetFood().

{

#ifdef \_\_CJTDebug\_5

if ( IsAlive() != 0x0DEADC0DE ) DEADCODEError();

#endif

double hind;

int rain = (int) m\_OurLandscape->SupplyRain()/2;

// Assume half of this falls at night

// Make sure the indices remain in bounds

if ( rain > 10 ) rain = 10;

int temp = (int) (floor( 0.5 + m\_OurLandscape->SupplyTemp() ));

if ( temp < 0 ) temp = 0;

if ( temp > 20 ) temp = 20;

hind = RainHindrance[rain];

hind \*= TempHindrance[ temp ];

return hind;

}

|  |  |  |  |  |  |
| --- | --- | --- | --- | --- | --- |
| double Skylark\_Adult::RemoveEM | ( | double | *food* | ) |  |

protectedvirtual

References Skylark\_Base::m\_OurPopulationManager, Skylark\_Base::m\_pcide\_conc, Skylark\_Base::m\_pesticide\_accumulation, and Skylark\_Population\_Manager::SupplyEMi().

Referenced by Skylark\_Female::FeedYoung(), Skylark\_Male::st\_CaringForYoung(), and Skylark\_Female::st\_PreparingForBreeding().

{

#ifdef \_\_CJTDebug\_5

if ( IsAlive() != 0x0DEADC0DE ) DEADCODEError();

#endif

// remove metabolic requirements

double used = m\_OurPopulationManager->SupplyEMi();

#ifdef \_\_PESTICIDE\_RA\_ADULT

// If using pesticide risk assessment then the total mg of pesticide collected that day are now in m\_pcide

// But, there well be more than we wanted, so now we take a mean as mg/kcal resource

m\_pcide\_conc = m\_pcide/food;

// Note there is an assumption here that they only eat insects. We know this to be incorrect so depending upon the

// aim of the assessment the line below may need to be modified (e.g. by scaling by the relative calorific content of

// insects compared to plant matter.

m\_pesticide\_accumulation = m\_pesticide\_accumulation+(m\_pcide\_conc\*(used));

#endif

food -=used;

if ( food < 0 ) food = 0;

return food; // Food still in gdw insects

}

---

## Member Data Documentation

bool Skylark\_Adult::BSuccess

protected

Referenced by Skylark\_Male::DefendTerritory(), Skylark\_Female::EstablishTerritory(), Skylark\_Female::OnBreedingSuccess(), Skylark\_Female::OnMaleNeverComesBack(), Skylark\_Male::OnMateDying(), Skylark\_Female::OnMateHomeless(), Skylark\_Male::OnMateLeaving(), Skylark\_Male::OnMateNeverComesBack(), Skylark\_Male::OnPairing(), Skylark\_Male::OnPreFledgelingMature(), Skylark\_Adult(), Skylark\_Male::Skylark\_Male(), Skylark\_Female::st\_Finding\_Territory(), Skylark\_Female::st\_GivingUpTerritory(), Skylark\_Male::st\_Immigrating(), Skylark\_Male::st\_ScaringOffChicks(), and Skylark\_Female::st\_StoppingBreeding().

int Skylark\_Adult::GoodWeather

protected

Reimplemented in Skylark\_Male.

Referenced by Skylark\_Adult(), Skylark\_Female::st\_Emigrating(), Skylark\_Female::st\_Flocking(), and Skylark\_Female::st\_TempLeavingArea().

vector<Point>\* Skylark\_Adult::m\_aTerrlist

protected

Referenced by Skylark\_Male::EstablishingATerritory(), Skylark\_Male::OnEvicted(), Skylark\_Adult(), Skylark\_Male::st\_Arriving(), Skylark\_Male::st\_FindingTerritory(), and ~Skylark\_Adult().

double Skylark\_Adult::MyExtractEff

protected

Referenced by Skylark\_Male::GetFood(), and Skylark\_Adult().

skTerritory\_struct Skylark\_Adult::MyTerritory

protected

Referenced by Skylark\_Male::ConstructAHabitatTable(), Skylark\_Male::EstablishingATerritory(), Skylark\_Female::EstablishTerritory(), Skylark\_Male::OnEvicted(), Skylark\_Female::OnMateDying(), Skylark\_Female::OnMateHomeless(), Skylark\_Male::OnMateLeaving(), Skylark\_Male::OnReHouse(), Skylark\_Male::ReEvaluateTerritory(), Skylark\_Adult(), Skylark\_Male::st\_Arriving(), Skylark\_Male::st\_AttractingAMate(), Skylark\_Female::st\_Dying(), Skylark\_Male::st\_Dying(), Skylark\_Male::st\_FindingTerritory(), Skylark\_Male::st\_FollowingMate(), Skylark\_Female::st\_GivingUpTerritory(), Skylark\_Female::st\_StoppingBreeding(), and Skylark\_Male::Supply\_Territory().

bool Skylark\_Adult::Paired

Referenced by Skylark\_Population\_Manager::BreedingPairsOutput(), Skylark\_Male::DefendTerritory(), Skylark\_Female::EstablishTerritory(), Skylark\_Female::OnBroodDeath(), Skylark\_Male::OnBroodDeath(), Skylark\_Female::OnClutchDeath(), Skylark\_Male::OnEvicted(), Skylark\_Female::OnFarmEvent(), Skylark\_Female::OnMaleNeverComesBack(), Skylark\_Male::OnMateDying(), Skylark\_Female::OnMateHomeless(), Skylark\_Male::OnMateLeaving(), Skylark\_Male::OnMateNeverComesBack(), Skylark\_Male::OnPairing(), Skylark\_Male::OnPreFledgelingMature(), Skylark\_Male::OnReHouse(), Skylark\_Adult(), Skylark\_Female::st\_Arriving(), Skylark\_Male::st\_Dying(), Skylark\_Female::st\_Finding\_Territory(), Skylark\_Male::st\_FollowingMate(), Skylark\_Female::st\_GivingUpTerritory(), Skylark\_Clutch::st\_Hatching(), Skylark\_Female::st\_Immigrating(), Skylark\_Male::st\_Immigrating(), Skylark\_Female::st\_StoppingBreeding(), Skylark\_Female::Step(), Skylark\_Population\_Manager::TheBreedingFemalesProbe(), Skylark\_Population\_Manager::TheCIPEGridOutputProbe(), Skylark\_Population\_Manager::TheNWordOutputProbe(), and Skylark\_Population\_Manager::TheRipleysOutputProbe().

---

The documentation for this class was generated from the following files:

- skylarks\_all.h
- skylarks\_all.cpp


- Skylark\_Adult
- Generated on Thu Jan 10 2013 13:15:37 for ALMaSS Skylark ODdox by
   1.8.1.1
